# *In vivo* genetic analysis of *Pseudomonas aeruginosa* carbon catabolic repression through the study of CrcZ pseudo-revertants shows that Crc-mediated metabolic robustness is needed for proficient bacterial virulence and antibiotic resistance

**DOI:** 10.1128/spectrum.02350-23

**Published:** 2023-10-30

**Authors:** Teresa Gil-Gil, José Ramón Valverde, José Luis Martínez, Fernando Corona

**Affiliations:** 1 Centro Nacional de Biotecnología, CSIC, Madrid, Spain; McGill University, Ste-Anne-de-Bellevue, Quebec, Canada

**Keywords:** *Pseudomonas aeruginosa*, antibiotic resistance, virulence, Crc, Catabolic repression, CrcZ

## Abstract

*Pseudomonas aeruginosa* is an opportunistic bacterial pathogen able to colonize a variety of habitats. Its success in colonizing these habitats relies on its metabolic robustness and its capability of efficiently using available carbon sources in a hierarchical way. *P. aeruginosa* carbon catabolic repression is post-transcriptionally regulated by Hfq and Crc, which form a complex that binds and impedes the translation of their target mRNAs. Under no catabolic repression conditions, the complex is sequestered by the small RNA CrcZ, allowing the translation of the involved mRNAs. In addition to regulating carbon sources use, Crc and Hfq modulate *P. aeruginosa* virulence and antibiotic resistance. In the absence of CrcZ, catabolic repression should be constitutive, severely impairing *P. aeruginosa* fitness. A Δ*crcZ* mutant was generated. As predicted, it presents severe fitness defects and alterations in virulence and antibiotic resistance. Pseudo-revertants that restore *P. aeruginosa* fitness, antibiotic resistance, and virulence were selected. Notably, most pseudo-revertants presented mutations in *crc*, despite Hfq, not Crc, being the RNA-binding protein of the complex. The analysis of several traits, including antibiotic resistance and bacterial virulence of these mutants, indicates that they can be grouped into two categories, those in which Crc is fully inactivated and those presenting smaller structural changes. The phenotypes of the latter resembling those of the wild-type strain. Notably, even when Hfq is not sequestered by CrcZ, in the Δ*crcZ* mutant, the lack of Crc impedes a proficient catabolic repression, indicating that Crc is strictly required for keeping *P. aeruginosa* metabolic robustness, virulence, and antibiotic resistance.

**Iimportance:**

Hfq and Crc regulate *P. aeruginosa* carbon catabolic repression at the post-transcriptional level. *In vitro* work has shown that Hfq binds the target RNAs and Crc stabilizes the complex. A third element in the regulation is the small RNA CrcZ, which sequesters the Crc-Hfq complex under no catabolic repression conditions, allowing the translation of the target mRNAs. A Δ*crcZ* mutant was generated and presented fitness defects and alterations in its virulence potential and antibiotic resistance. Eight pseudo-revertants that present different degrees of fitness compensation were selected. Notably, although Hfq is the RNA binding protein, most mutations occurred in Crc. This indicates that Crc is strictly needed for *P. aeruginosa* efficient carbon catabolic repression *in vivo*. The compensatory mutations restore in a different degree the alterations in antibiotic susceptibility and virulence of the Δ*crcZ* mutant, supporting that Crc plays a fundamental role linking *P. aeruginosa* metabolic robustness, virulence, and antibiotic resistance.

## INTRODUCTION

*Pseudomonas aeruginosa* is a ubiquitous bacterial species able to colonize a large set of ecosystems (1, 2). Among them, this microorganism is capable of colonizing the human body, being one of the most important opportunistic pathogens causing infections at hospitals as well as in patients suffering from chronic obstructive pulmonary disease or cystic fibrosis (3 – 5). Part of the success of *P. aeruginosa* for growing in such a diverse number of habitats is its capability of using different nutrient sources in a tightly regulated manner. Indeed, *P. aeruginosa* possesses in its genome a large number of regulators—including RNA regulators (6)—and sensor systems that allow this microorganism to trigger specific metabolic responses when confronted with different signals and nutritional situations (7). One of the situations that microorganisms have to solve is their response to the presence of different nutrients when growing in complex habitats. A common mechanism to address this issue is the hierarchical assimilation of nutrients, through the sequential activation of the catabolic pathways of the preferred nutrient sources and the repression of the other pathways until the preferred nutrients disappear, which has been named as catabolite repression (8). It was early proposed that, in *Pseudomonadaceae*, carbon catabolite repression is governed by the catabolite repression control protein Crc (9). However, further work showed that Crc lacks RNA-binding activity, being that the RNA chaperon Hfq is the protein that actually binds the proposed RNA targets (10, 11). While it has been discussed that upon this situation, Crc should have an “ancillary” role and the actual regulator would be Hfq, it is known that Crc-deficient mutants present several different phenotypic changes and alterations in their proteome (12, 13) in a Hfq wild-type context. This indicates that, despite its inability of binding RNA, Crc has a critical role in the regulation of the hierarchical utilization of carbon sources in *Pseudomonas* (14, 15), as well as in other aspects of relevance for the physiology of *Pseudomonas aeruginosa* as virulence, antibiotic resistance, or response to oxidative stress (16 – 18). It is relevant noticing that Hfq homologs are present in several bacteria that do not present this catabolic repression regulatory pathway. Actually, Hfq is considered as an RNA chaperon (19) that promotes interactions between small non-coding RNAs (sRNAs) and mRNAs. In the case of *P. aeruginosa*, recent work has shown that, besides catabolic repression, Hfq is involved in several processes that go beyond catabolic repression and that the effect of Hfq on *P. aeruginosa* physiology can be strain specific (20). Altogether, these data indicate that Hfq is not a specific, independent regulator. It is instead the required RNA chaperon needed for regulating, together with other proteins (as Crc) or sRNAs, a variety of post-transcriptionally regulated bacterial processes.

Further *in vitro* work showed that Hfq and Crc form a tertiary complex with their target mRNAs (21, 22), in which Hfq binds the mRNAs and Crc facilitates the formation of a more stable complex at these targets (23, 24). The complex is composed of two Hfq hexamers, two Crc molecules, and two molecules of the target RNA (22). A third element in the system consists on the Crc-associated small RNA CrcZ, which contains five Hfq/Crc-binding motifs (25). When expressed at high levels, CrcZ sequesters Hfq/Crc, and catabolite repression is not achieved. It is relevant to notice that the concentrations of Hfq and Crc do not present substantial changes depending on the nutrients’ availability (10, 25), whereas CrcZ concentrations are different depending in the carbon sources, low at conditions that trigger carbon catabolite repression and high when carbon catabolite repression is released (25). Regulation of expression in response to environmental changes requires changes in the action of the regulators involved, either in their activity or in their concentration. Since among the three elements (Crc, Hfq, and CrcZ) involved in carbon catabolite control, only CrcZ presents alterations in its concentration, this small RNA is central to the *P. aeruginosa* response to changes in carbon sources’ availability. When CrcZ is absent, it could be predicted that *P. aeruginosa* should face a hyper-repressed phenotype for the use of carbon sources. This situation will impede *P. aeruginosa* from making a proficient use of nutrients, and consequently, *crcZ*-defective mutants should be strongly impaired for growth and hence genetically unstable; consequently, compensatory mutations could be selected. In the present work, we took advantage of this situation and searched for spontaneous mutants able to compensate the physiological impairment potentially displayed by a *crcZ P. aeruginosa*-deficient mutant. Classical genetic studies, based on the analysis of suppressor mutations, mainly when the mutations occur in a different gene (pseudo-revertants), have demonstrated to be excellent tools for determining the functional linkage of several bacterial elements, and the use of whole-genome sequencing approaches may serve to delimit the suppressor mutations involved. In the current work, we have used this approach for a functional *in vivo* characterization of the elements genetically linked to CrcZ, a main player in *P. aeruginosa* carbon catabolite repression.

## RESULTS AND DISCUSSION

As stated in the introduction, Crc was firstly described as a key element for the regulation of the use of preferential carbon sources when *P. aeruginosa* grows in complex media (26). More recent work indicates that Crc also modulates antibiotic resistance and virulence, as well as the production of vesicles in this bacterial pathogen (12, 17, 27), being a central element in keeping *P. aeruginosa* metabolic robustness (18). With the aim of increasing our knowledge on the crosstalk among metabolic regulation and the virulence and antibiotic resistance of bacterial pathogens, we generated a deletion mutant in *crcZ* (Table 1). This small RNA is the key element modulating carbon catabolic repression in *P. aeruginosa*, since its intracellular concentration changes as a function of the presence of different carbon sources in the environment. Unfortunately, the mutant presented unstable phenotypes (see below), a feature that may compromise its use for the analysis of the role of CrcZ in regulating virulence and resistance in *P. aeruginosa*. Nevertheless, we took advantage of this situation for analyzing in detail the mutations that restore the fitness of the ∆*crcZ* mutant to levels similar to those of the wild type in the aim of delimitating more precisely, genetically, and *in vivo* the regulatory network involved in carbon catabolite repression in *P. aeruginosa*.

**TABLE 1:** Strains and plasmids used in this study

Strain or plasmid	Relevant features	Origin
*P. aeruginosa*		
PAO1/Wt	Wild-type strain of *P. aeruginosa*. Conserved by P. V. Phibbs.	(28)
FCP001/Δ*crc*	PAO1, Δ*crc*	(12)
FCP002/Δ*crcZ*	PAO1, Δ*crcZ*	This work
Δ*crcZ*_e1, e2, e3, e5	FCP002, Δ*crcZ,* FAA^R^	This work
Δ*crcZ*_r1, *r2*, r4, r5	FCP002, Δ*crcZ,* FAA^S^	This work
*E. coli*		
OP50	Used for the feeding of *C. elegans*	(29)
S17-1	Hosting and mobilizing strain in conjugations. F− *recA1 endA thiE1 pro-82 creC510 hsdR17* RP4-Tc::Mu-Km::Tn7	(30)
One Shot Top10	Used for cloning purposes. F− *mcrA* Δ(*mrr-hsd*RMS-*mcr*BC) Φ80*lac*ZΔM15 Δ *lac*X74 *rec*A1 *ara*D139 Δ(*araleu*)7697 *gal*U *gal*K *rps*L (StrR) *end*A1 *nup*G	Invitrogen
		
Plasmid		
pGEM-T easy	Cloning vector; *P_sp6_ lacZ lacO P_T7_ oriV*(pMB1) *ori*(f1) *bla*; Amp^R^	Promega
pEX18Ap	Used for homologous recombination; Amp^R^-Cb^R^	(31)
pGEM-T-Δ*crcZ*	pGEM-T with 2,000 pb of the *crcZ* flaking regions	This work
pEX18Ap-Δ*crcZ*	pEX18Ap with 2,000 pb of the *crcZ* flaking regions	This work

### A *P. aeruginosa* ∆*crcZ* mutant is less proficient for using non-preferential carbon sources than the wild-type strain

A ∆*crcZ* mutant was obtained from *P. aeruginosa* PAO1 by homologous recombination as described in Materials and Methods. The deletion of *crcZ* was confirmed using the primers crcZ_F_HindIII, crcZ_R_HindIII, crcZ_ver_F, and crcZ_ver_R (Table 2). The current model of *P. aeruginosa* carbon catabolite repression states that CrcZ titrates Crc/Hfq when repression is not needed (21, 25, 32). Consequently, the lack of CrcZ will produce the constitutive repression of the pathways regulated by Crc/Hfq independently on the carbon source present in the medium. To address this issue, we analyzed the effect of deleting *crcZ* on *P. aeruginosa* growth in a rich medium (lysogeny broth [LB]) and in a minimal medium (M63) in the presence of preferential carbon sources (succinate) and non-preferential carbon sources (gluconate, citrate, and mannitol).

**TABLE 2:** Primers used in this study

Primer name	Sequence 5′−3′	Use
*crcZ*_F_HindIII	CCAAAGCTTGGGCTGATCGAATCCGAGCTGTT	*crcZ* deletion
*crcZ*_F_cons_R	AGGCGAAGAAAACGGGTTGTTGTGCCAATACATA	*crcZ* deletion
*crcZ*_R_HindIII	CCAAAGCTTGGGGCACCGGGGTCTTCCAGATAAC	*crcZ* deletion
*crcZ*_R_consR	TATGTATTGGCACAACAACCCGTTTTTCCTTCGCCT	*crcZ* deletion
*crcZ*_ver_F	CGGGCTTGTTGTTTTTGTTT	Δ*crcZ* verification
*crcZ*_ver_R	CAAGCAACGACGAAGACAAT	Δ*crcZ* verification

**Fig 1:**
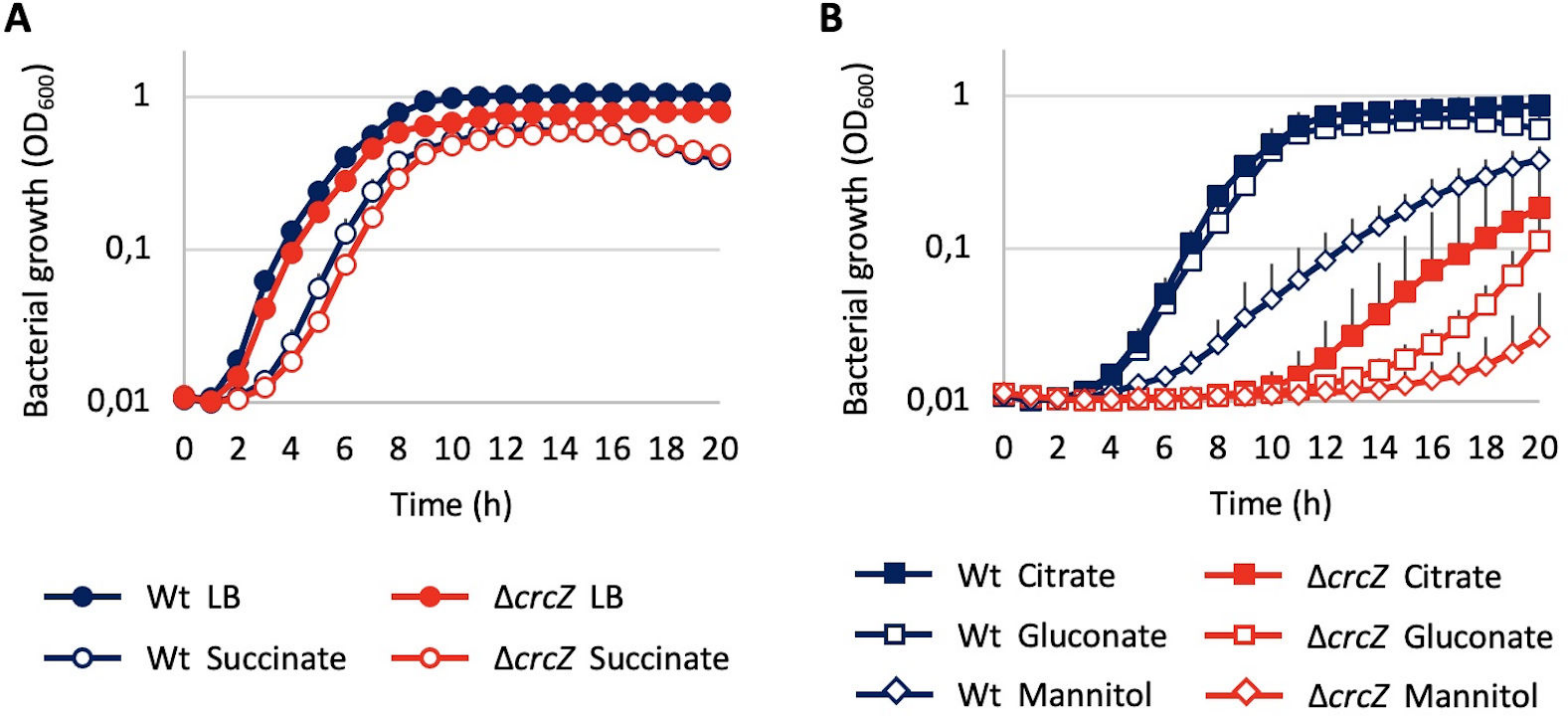
Growth of PAO1 and ∆*crcZ* strains in different media. (**A**) Growth in LB and in M63 with succinate at 40 mM. (**B**) Growth in M63 with citrate, gluconate, or mannitol at 40 mM. Experiments were performed in triplicate; error bars represent standard deviation.

As shown in Fig. 1, while the growth of the mutant is not impaired when growing in preferential carbon sources, whose utilization is not precluded by catabolite repression, its growth is impaired when growing using secondary carbon sources. This information is consistent with the statement that the expression of the catabolic pathways of these carbon sources would be repressed when CrcZ is absent. Although a decrease in the optical density at which the ∆*crcZ* mutant enters in stationary phase is observed when it grows in LB, the effect is not as drastic as that observed in the presence of non-preferential carbon sources. This feature is consistent with the presence of both preferential and non-preferential carbon sources in rich media as LB.

### A *P. aeruginosa* ∆*crcZ* mutant presents altered virulence and is less susceptible to antibiotics than the wild-type strain

**Fig 2:**
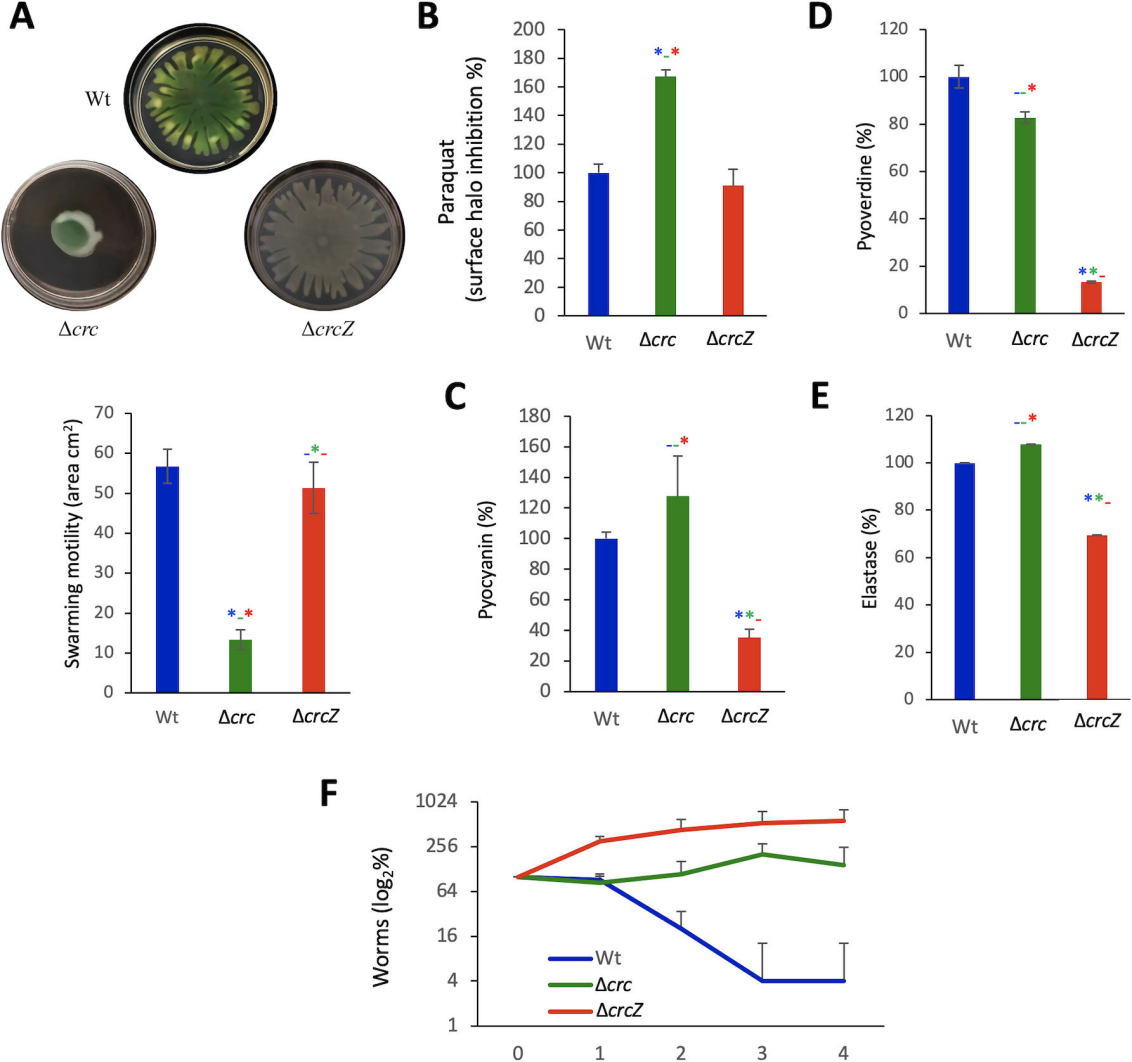
The lack of either *crc* or *crcZ* alters *P. aeruginosa* virulence (**A**). Swarming motility of PAO1, ∆*crc*, and ∆*crcZ*. Ten microliters of an overnight culture of such strains were spotted in an LB medium with agar at 0.50% Petri dish. Maximum diameters of the growing colonies were measured after 20 h at 37°C. Representative plates are shown. (**B**) To measure the susceptibility to paraquat, a sterile disk with 1 µmol of paraquat was dropped in a plate seeded with the selected strains. After 1 day, the growth inhibition halo surface was measured. Experiment was performed in triplicate. (**C**) Pyocyanin, (**D**) pyoverdine, and (**E**) elastase productions were measured in triplicate as described in Materials and Methods. Results were normalized taking into consideration the OD_600_ of the cultures and represented as percentage of the value of the wild-type strain. Error bars represent standard deviation. (**F**) Virulence assays with *C. elegans* N2. Experiment was performed with five replicates; error bars represent standard deviations. Asterisk indicates *P* < 0.05 calculated by unpaired two-tail *t*-test. Blue, comparison with the wild-type strain; green, comparison with the ∆*crc* mutant; and red, comparison with the ∆*crcZ* strain.

Previous work has shown that the absence of Crc makes *P. aeruginosa* more susceptible to antibiotics and less virulent than its parental strain when growing in a rich medium that contains a variety of substrates (17). It could then be expected that the ∆*crcZ* mutant should present opposite phenotypes. In order to compare our results with previous data published in the field, we used the standard growing conditions (rich media in all cases) for each of the tested phenotypes. As shown in Table 3, the ∆*crcZ* mutant was more resistant to several of the antibiotics to which the ∆*crc* mutant was more susceptible. When looking to elements relevant for *P. aeruginosa* virulence, we found that, while the lack of Crc strongly reduces *P. aeruginosa* motility, the effect on swarming motility was more subtle in in the case of the ∆*crcZ* mutant (Fig. 2A). As can be seen, the dendritic pattern was not exactly the same in both strains, but the surface covered did not present statistically significant differences. In agreement with previous work (18), the ∆*crc* mutant presents an impaired response to oxidative stress (Fig. 2B). However, no significant differences in the susceptibility to paraquat were observed for the ∆*crcZ* mutant. Noteworthily, this mutant presents an impaired pyocyanin production, a phenotype opposite to the one observed for the *crc*-defective mutant (33) (Fig. 2C). Elastase and pyoverdine productions were also impaired in the ∆*crcZ* mutant, while no significant differences were observed between the wild-type strain PAO1 and the ∆*crc* mutant. This might imply that the lack of CrcZ modifies the virulence phenotype of *P. aeruginosa*. To address this possibility, we used a *C. elegans* virulence model. Notably, the ∆*crcZ* mutant presented an impaired virulence phenotype, being even less virulent than the *crc* mutant (Fig. 2D). These results indicate that bacterial virulence requires a well-balanced bacterial metabolism and its misbalance in any of both directions driven by the lack of Crc (no catabolite repression) or of CrcZ (constitutive catabolite repression) may render the same hypo-virulent phenotype.

**TABLE 3:** Susceptibility to antibiotics of the strains analyzed in the work

Strain	Minimal inhibitory concentrations (µg/mL)				
	Fosfomycin	Imipenem	Kanamicin	Amikacin	Ofloxacin
PAO1	64	3	8	2	0.5
PAO1 Δ*crc*	32	1	4	1	0.38
PAO1 Δ*crcZ*	384	1	32	6	0.75
R1	32	0.5	6	1	0.38
R2	32	1	6	1	0.38
R4	32	1	6	1	0.5
R5	48	1	6	1	0.38
E1	64	1.5	16	2	0.5
E2	64	1.5	16	2	0.75
E3	192	2	24	3	0.75
E5	192	0.5	8	2	0.5

### A ∆*crcZ* mutant is unstable when growing in rich LB broth

**Fig 3:**
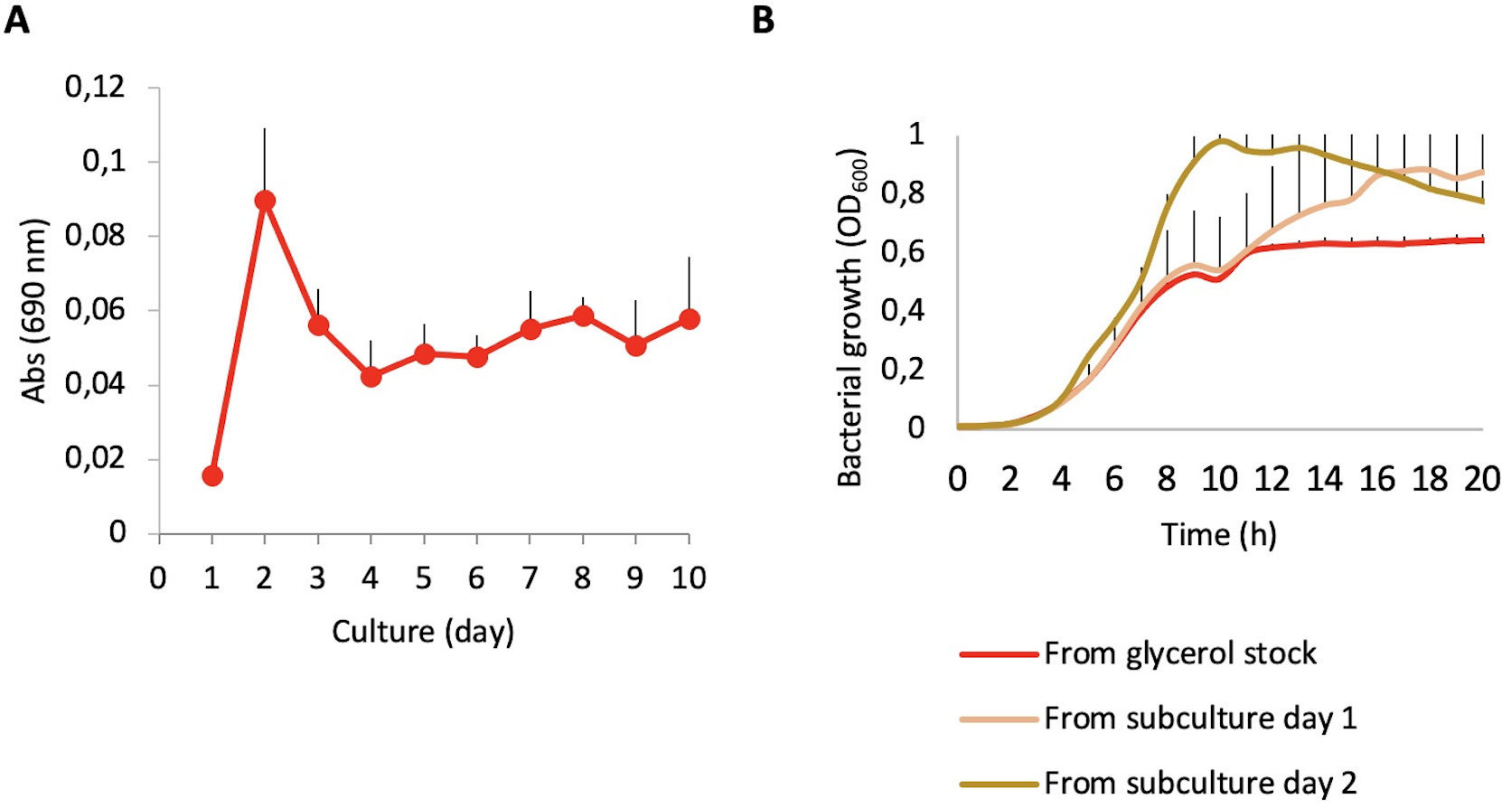
The phenotype of the ∆*crcZ* mutation is unstable. (**A**) Pyocyanin production measured each day of a ∆*crcZ* culture. Absorbance at 690 nm was recorded of the supernatant of five biological replicates. Error bars represents standard deviation. (**B**) Bacterial growth curves of different subcultures of a ∆*crcZ* strain. Values are the average of three replicates, and error bars represent standard deviation.

We have observed that when the ∆*crcZ* mutant grows in minimal medium plates containing non-preferred carbon sources, such as alanine, some large colonies appear (not shown), likely indicative of the selection of suppressor mutants in these stringent growing conditions. In addition, we have observed that the ∆*crcZ* mutant presents alterations in its resistance to antibiotics and virulence even when growing in LB, a rich, complex medium with a variety of carbon sources (14, 15). This suggests that, even when growing in complex media, the ∆*crcZ* mutant may have fitness defects. Indeed, we found that, although clear phenotypes are observed when the ∆*crcZ* mutant is grown from stock, the situation changes when the mutant is subjected to sequential subcultures even in LB medium (Fig. 3). In other words, in a rich media, which present an abundance of primary and secondary carbon sources and hence constitutive catabolic repression driven by the absence of CrcZ should not be deleterious, the lack of this small RNA may compromise *P. aeruginosa* fitness in such a way that compensatory mutations can be selected upon evolution in this rich medium.

For example, as shown in Fig. 3A, the production of pyocyanin changes along 7 days of subculture, suggesting the possibility of an enrichment of mutants able to compensate the fitness cost that the ∆*crcZ* mutant presents. In favor of this possibility is the finding that, after 2 days of sequential passage, the ∆*crcZ* mutant presents an improved growth in LB as compared with the initial mutant (Fig. 3B). It is then possible that the sequential subculture of the ∆*crcZ* mutant allows the enrichment of mutants containing suppressor mutations of the *crcZ* deletion. Since the diversity and frequency of these mutants would be different to the ones that appear when Δ*crcZ* grows in minimal medium in presence of secondary carbon sources, analyzing these mutants that arise at mild selection conditions is of relevance in order to identify both mutants that fully abolish catabolite repression and mutants that abolish this repression just in part. Given that the *crcZ* gene is absent, reversion to the original genotype is not possible; mutations must be pseudo-revertants, laying in genes coding elements functionally linked to CrcZ.

### Isolation of ∆*crcZ* pseudo-revertants

Previous work aiming at identifying ∆*crc* pseudo-revertants was based on the use of fluoroacetamide (FAA) as selective agent. The *P. aeruginosa* AmiE enzyme transforms FAA into fluoroacetate, which is toxic when it is transformed into fluorocitrate (34). The expression of AmiE is under catabolite repression; when *P. aeruginosa* grows using succinate as the carbon source, AmiE is not produced, and FAA is not toxic. Nevertheless, if the regulation involved in catabolite repression is absent, AmiE is produced in all conditions, and FAA is toxic. Consistent with such situation, FAA is toxic when the ∆*crc* mutant grows in succinate plus FAA. In other words, FAA susceptibility phenotype is a proxy of the catabolite repression phenotype. To isolate pseudo-revertant mutants presenting different levels of catabolic repression, five different replicates of the ∆*crcZ* mutant were grown in LB broth, with daily subcultures along 10 d and 20 colonies were isolated from each bacterial population.

**Fig 4:**
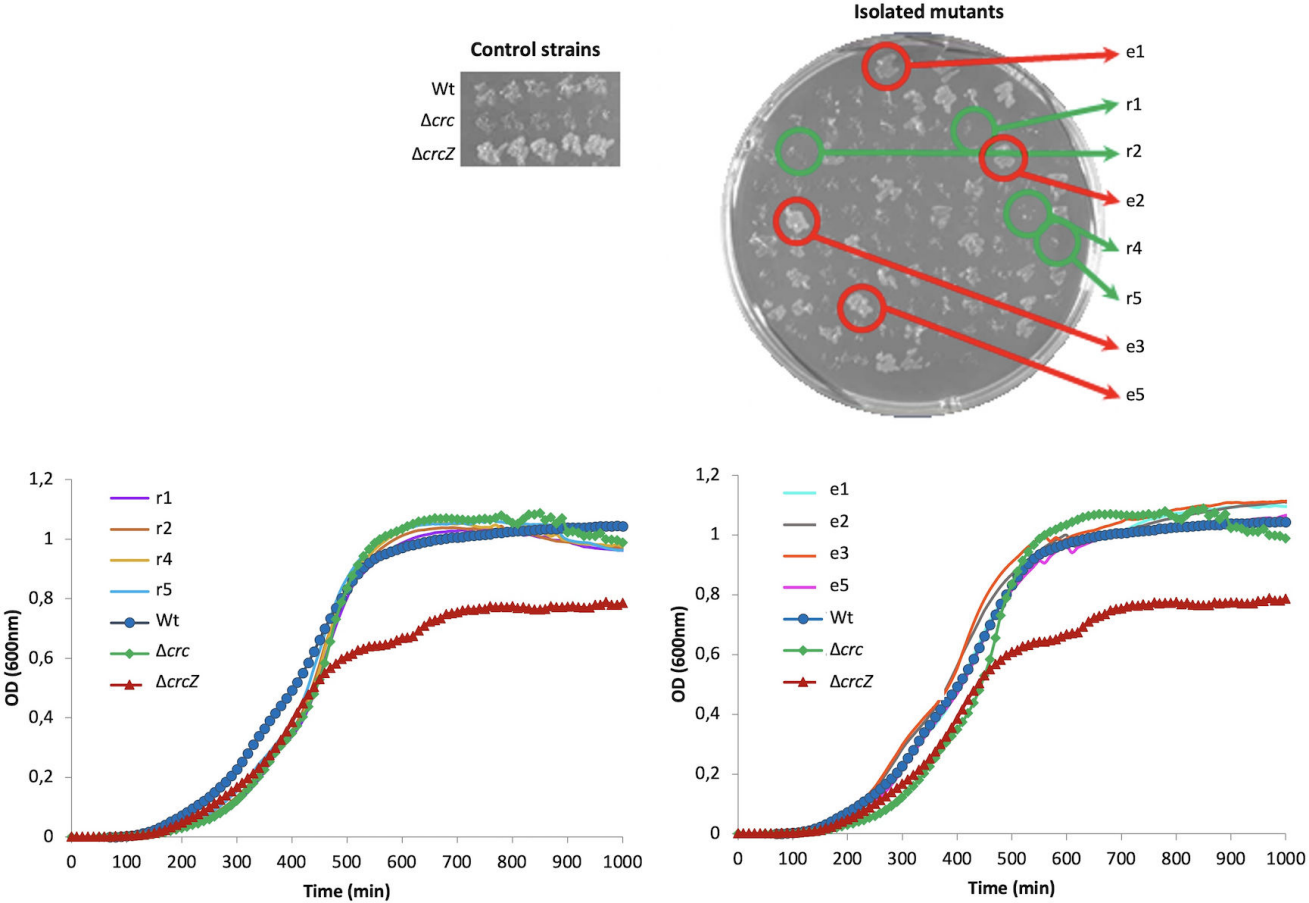
Selection of Δ*crcZ* pseudo-revertants. FAA phenotype of 100 colonies. Twenty colonies of five replicates of the Δ*crcZ* sixth subculture were streaked in M63 agar plate plus succinate at 40 mM and FAA at 5 mg/mL. Plates were incubated at 37°C during 24 h. The growth of the pseudo-revertants in LB was measured. As shown, in all cases, the mutants recover the growth of the wild-type strain.

The colonies were reseeded in M63 plates containing succinate as carbon source and FAA, and the growth of the colonies was recorded after 24 h of incubation. As shown in Fig. 4, we could distinguish isolates that poorly grew under these conditions, which most likely had lost AmiE catabolite repression (∆*crcZ*-r mutants) and strains that grew as the wild-type or the ∆*crcZ* mutant strains (∆*crcZ*-e mutants). We picked up four isolates from each of the categories for further analysis. As shown in Fig. 4, while the ∆*crcZ* presented an impaired growth in LB, the growth of the mutants was similar to that of the wild-type strain.

### Mutations in either *crc* or *hfq* revert, in different degree, the phenotype of the ∆*crcZ* mutant

To identify the mutations involved in the reversion of the phenotype of the ∆*crcZ* mutants, the genomes of previously isolated mutants were sequenced. Only those mutants unable to grow competently on FAA and using succinate as a carbon source could be expected to have mutations in elements involved in catabolic repression. However, we detected that all mutants presented this type of mutations; in all cases except one, which presents a mutation in *hfq*, mutations were detected in *crc*. The mutations of eight mutants, chosen for further studies, were confirmed by amplifying the corresponding genomic region and Sanger sequencing. In all cases, the genes only contained the mutations described in Table 4. While, as expected, the ∆*crcZ*-r mutants did not grow in succinate + FAA, the ∆*crcZ*-e mutants were able to grow under these conditions, presenting different duplication times. This indicates that the evolved ∆*crcZ* populations accumulate mutations that modulate in a different degree *P. aeruginosa* catabolite repression. Notably, most mutations occur in *crc*, indicating that Crc is a main player in *P. aeruginosa* catabolite repression and that likely, its mutation is less deleterious than the mutation of *hfq*.

**TABLE 4:** Mutations that revert the phenotype of the ∆*crcZ* mutant

Strain	Phenotype	Duplication time in Suc + FAA (min)	Mutations	Amino acid change
Δ*crcZ*_r1	FAA^S^	−^*a*^	6002185 G > A	Crc Trp22stop
Δ*crcZ*_r2	FAA^S^	−	6002563 C > T	Crc S148L
Δ*crcZ*_r4	FAA^S^	−	6002794 C > −	Crc G227fs
Δ*crcZ*_r5	FAA^S^	−	6002759–6002818 del	Crc Δ215-234
Δ*crcZ*_e1	FAA^R^	70 ± 1	6002680 G > C	Crc A188P
Δ*crcZ*_e2	FAA^R^	63 ± 2	6002589 G > A	Crc D157N
Δ*crcZ*_e3	FAA^R^	49 ± 1	6002793 A > C	Crc T225P
Δ*crcZ*_e5	FAA^R^	68 ± 2	5548480 A > T	Hfq Y55N

^*a*^ -: The mutant does not grow in Suc+FAA.

The mutations present in r1, r4, and r5 produce strong alterations in Crc primary sequence, hence abolishing its activity. However, the other mutations are alterations in single, specific amino acids, likely reducing the activity of the protein but not fully abolishing its function. In favor of this statement is the phenotype of these strains regarding their growth in presence of FAA (Table 4). To gain some insight in the consequences of the observed mutations in the function of Crc or Hfq, their structure was modeled.

**Fig 5:**
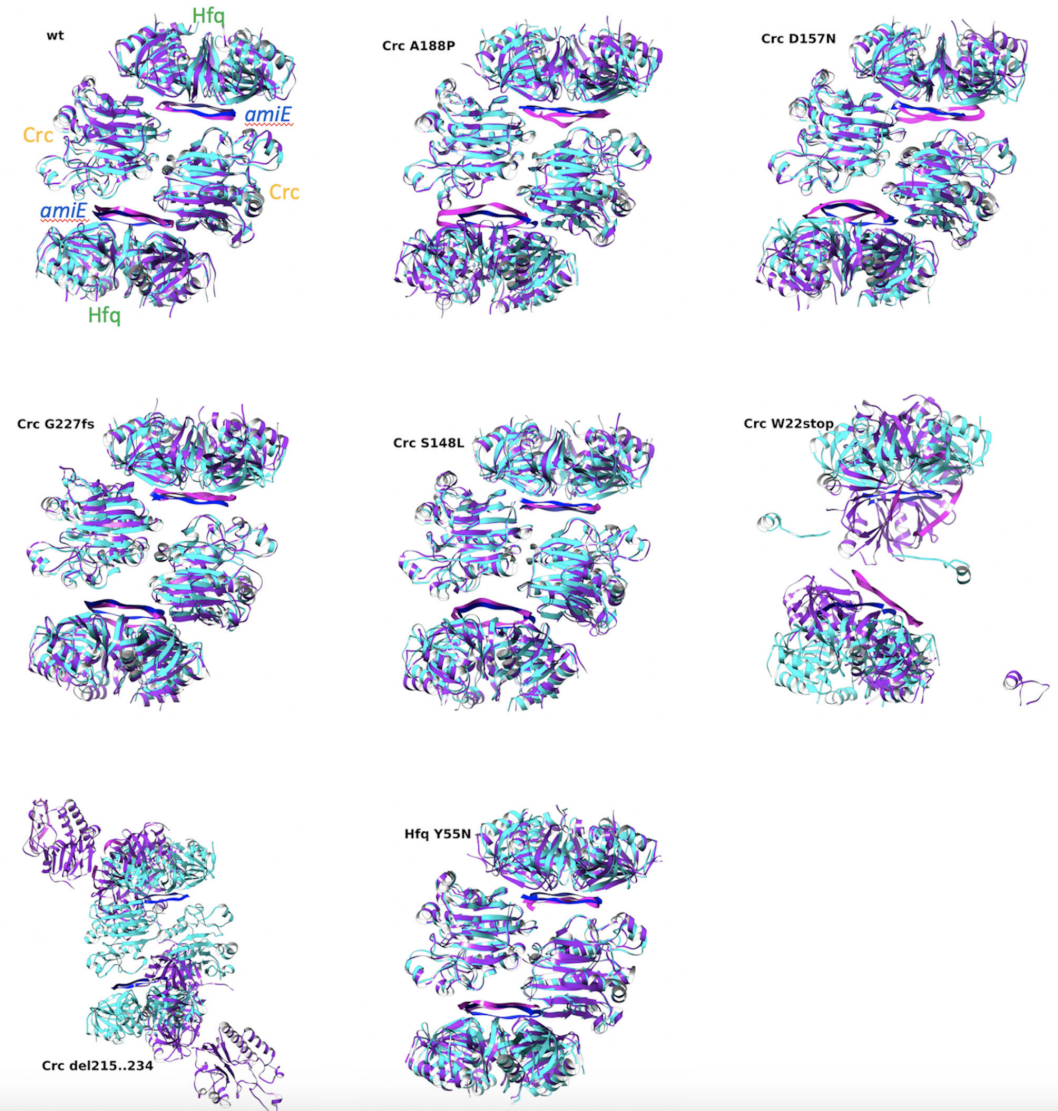
Molecular dynamics analysis of changes in the Crc-Hfq-*amiE* complex in the different ∆*crc* pseudo-revertants. The complex is formed by Hfq hexamers, two Crc molecules, and two molecules of *amiE* (22). While relevant changes in the structures were not seen, except in the inactivating mutations W22stop and Δ215-234, the complexes presented some degree of instability as compared with the wild type after MD simulation. In agreement with the functional information (see text), this supports that the mutants presented an impaired capacity of interacting with their RNA targets and proficiently regulated carbon catabolic repression. Cyan (protein) and blue (RNA): minimized structure prior to simulation. Magenta (protein) and pink (RNA): structure after MD simulation.

Molecular modeling of the mutants resulted in all cases in structures that could directly be substituted in the reference wild-type structure without clashes. Although some minor deviations from the reference structure could be visually identified in the initial models, no obvious structural differences that could directly explain the mutant phenotype were initially apparent except for mutants W22stop and Δ215-234, which showed clear differences for obvious reasons. After a molecular dynamics (MD) simulation of 110 ns, most of the complexes showed a significant instability. Superposed images of the mutant complexes prior and after simulation are provided in Fig. 5. Some of the models did, however, display distorted conformations in their final structure, most likely due to mutual adaptation of the components.

To better understand the impact of each mutation, we analyzed the interactions between the complex components before and after the MD simulation. The results are summarized in Table 5. Interestingly, mutations affecting Crc had little impact on the interactions between Hfq and *amiE* but did show different degrees of impact in the Crc-*amiE* and Crc-Crc interactions. The Hfq mutation, on the other hand, displayed a strong impact on the interaction with *amiE* and the Crc dimer, reflected in an initial absence of the H-bonds required for the initial formation of the complex.

**TABLE 5:** Interactions between the complex components before and after MD simulation

	Hfq-*amiE*		Hfq-Crc		Crc-Crc		Crc-*amiE*	
	H-bonds	Contacts	H-bonds	Contacts	H-bonds	Contacts	H-bonds	Contacts
WT (t0)	79	1009	9	81	14	76	36	318
WT (t1)	62	854	11	98	4	35	30	265
Crc A188P (t0)	78	1024	7	81	6	79	22	263
Crc A188P (t1)	47	680	14	112	8	72	33	328
Crc D157N (t0)	75	996	9	88	3	28	22	165
Crc D157N (t1)	48	643	18	153	14	101	21	257
Crc G227fs (t0)	75	1017	9	72	3	46	18	202
Crc G227fs (t1)	58	873	9	88	1	11	21	188
Crc S148L (t0)	76	1037	9	72	0	16	14	208
Crc S148L (t1)	59	802	14	109	7	63	25	228
Crc W22stop (t0)	74	989	0	0	0	0	0	0
Crc W22stop (t1)	71	930	1	45	0	0	0	0
Crc Δ215–234 (t0)	78	1063	11	69	0	0	23	252
Crc Δ215–234 (t1)	57	760	13	133	0	0	28	310
Hfq Y55N (t0)	55	680	3	40	13	80	36	332
Hfq Y55N (t1)	59	819	4	74	5	66	28	275

^*a*^ Counts of hydrogen bonds (H-bonds) and atomic contacts (Contacts) at each of the intermolecular surfaces in the wild-type and mutant 2:2:2 complexes, as observed before and after the molecular dynamics simulation.

The existing 2:2:2 complex for Hfq:Crc:*amiE* was initially selected because its assembly is the first step in the regulation process (22). Since visual inspection of MD trajectories is unreliable, unless very obvious changes can be detected, we decided to analyze the interactions at the contact interfaces. Interpreting the MD results requires comparison with the wild type at corresponding times: generally ,the wild type showed a strengthening of the interactions after the simulation, and a similar improvement could be seen to different degrees in the mutants, likely due to hydrophobic interactions and accommodation rearrangements induced by the solvent.

Regarding Crc, interpretation of mutant W22stop is trivial as this mutation removes in Crc all known amino acids involved in interactions with other members of the complex. Mutant A188P may reduce the number of H-bonds initially formed by Crc with Hfq, the other Crc subunit, and, significantly, *amiE*, suggesting an impact in the formation of the initial complex. Although it may eventually stabilize some interactions, it would lead to an unstable complex with a significantly weaker interaction of Hfq with *amiE*. D157N might have a significant impact on the Crc dimer and its interaction with *amiE*, which likely would lead to a weaker interaction with *amiE* of both Crc and Hfq. While, surprisingly, Crc G227fs might have a similar initial effect on the complex to D157N, it may ultimately result in a stronger reduction in the stability of the Crc dimer and its interaction with *amiE*. Curiously, S148L would have an even stronger effect on the constitution of the complex than G227fs, a somewhat smaller but still large impact on sustained interaction between Crc and *amiE*. Δ215-234 seems to preclude formation of the Crc dimer, although then it may lead to constitution of an unstable dysfunctional complex.

Regarding the Hfq mutant, the initial interaction with *amiE* and Crc is significantly reduced, suggesting that the mutation hampers the initial formation of the complex coupled with a largely sustained debilitation of its interaction with Crc, which further supports a potential impediment to the formation of stable complexes. In addition, the mutant may also weaken sustained interactions between Crc and *amiE*.

It is worth noting that early effects in the formation of the 2:2:2 complex likely have a stronger impact in the signaling process by affecting subsequent steps, and even if similarly strong interactions can be restored after the MD simulation, these are usually distorted, weaker than those reached by the wild type and would be likely unable to recover the function. As the analysis of the 2:2:2 complex proved sufficient to explain the effect of the mutations studied, simulating the dynamic recruitment process to the 2:3:2 and 2:4:2 complexes was not further pursued.

### Effect of the mutations that revert ∆*crcZ* on *P. aeruginosa* phenotypes regulated by Crc

To further correlate the observed mutations with changes in the phenotypes under catabolite repression control, we measured a set of these phenotypes in the selected mutants, with a particular focus on those phenotypes with relevance when *P. aeruginosa* is producing an infection.

**Fig 6:**
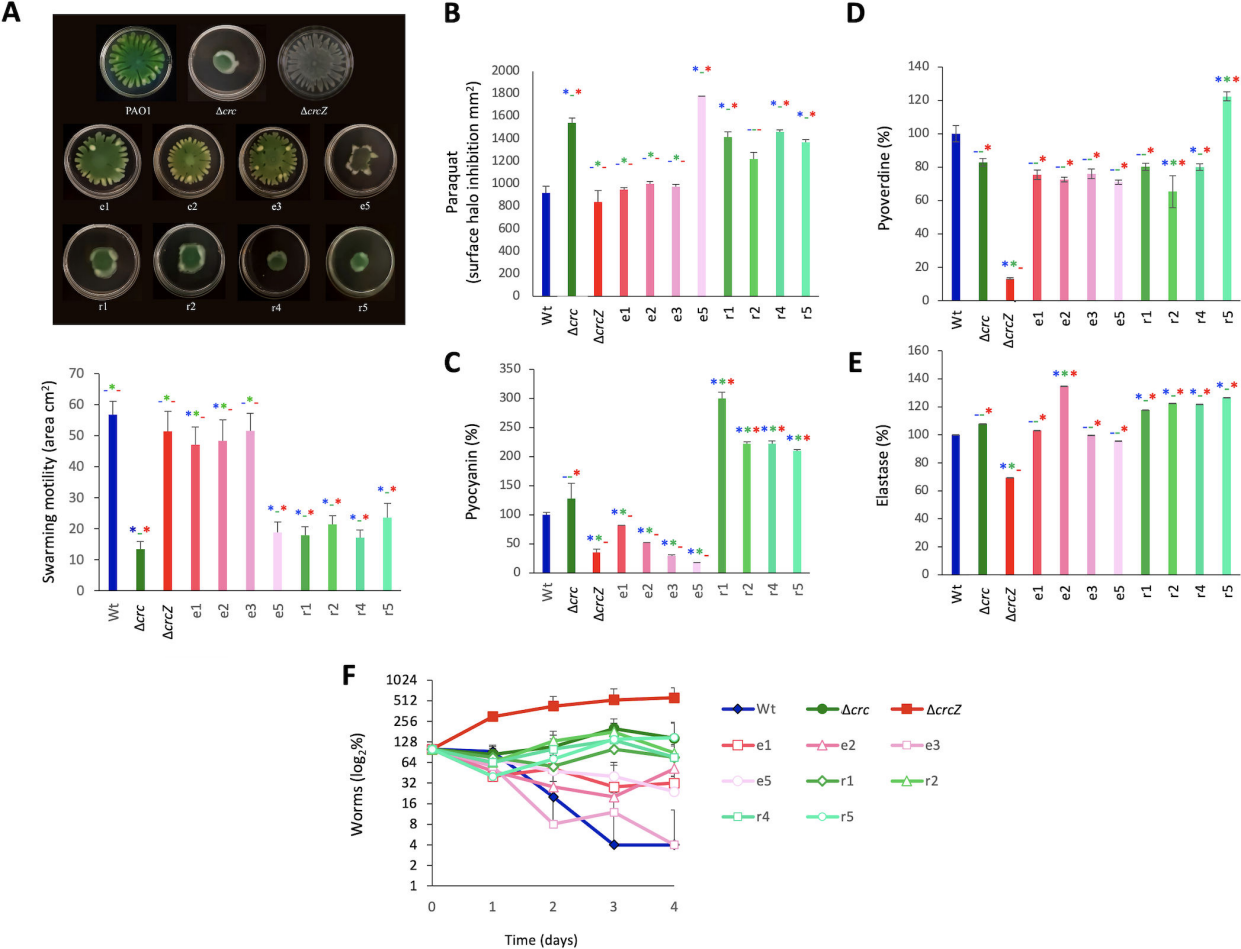
Phenotypic analysis of the *ΔcrcZ* pseudo-revertants. The experimental conditions are the same previously described in Fig. 2. (**A**) Swarming motility of PAO1, *Δcrc*, and *ΔcrcZ*. Ten microliters of an overnight culture of such strains were spotted in an LB medium with agar at 0.50% Petri dish. Maximum diameters of the growing colonies were measured after 20 h at 37°C. Representative plates are shown. (**B**) To measure the susceptibility to paraquat, a sterile disk with 1 µmol of paraquat was dropped in a plate seeded with the selected strains. After 1 day, the growth inhibition halo surface was measured. Experiment was performed in triplicate. (**C**) Pyocyanin, (**D**) pyoverdine, and (**E**) elastase productions were measured in triplicate as described in Materials and Methods. Results were normalized taking into consideration the OD_600_ of the cultures and represented as percentage of the value of the wild-type strain. Error bars represent standard deviation (**F**). Virulence assays with *C. elegans* N2. Experiment was performed with five replicates; error bars represent standard deviations. Asterisk (*) indicates *P* < 0.05 calculated by unpaired two-tail *t*-test. Blue, comparison with the wild-type strain; green, comparison with the ∆*crc* mutant; and red, comparison with the ∆*crcZ* strain.

**Fig 7:**
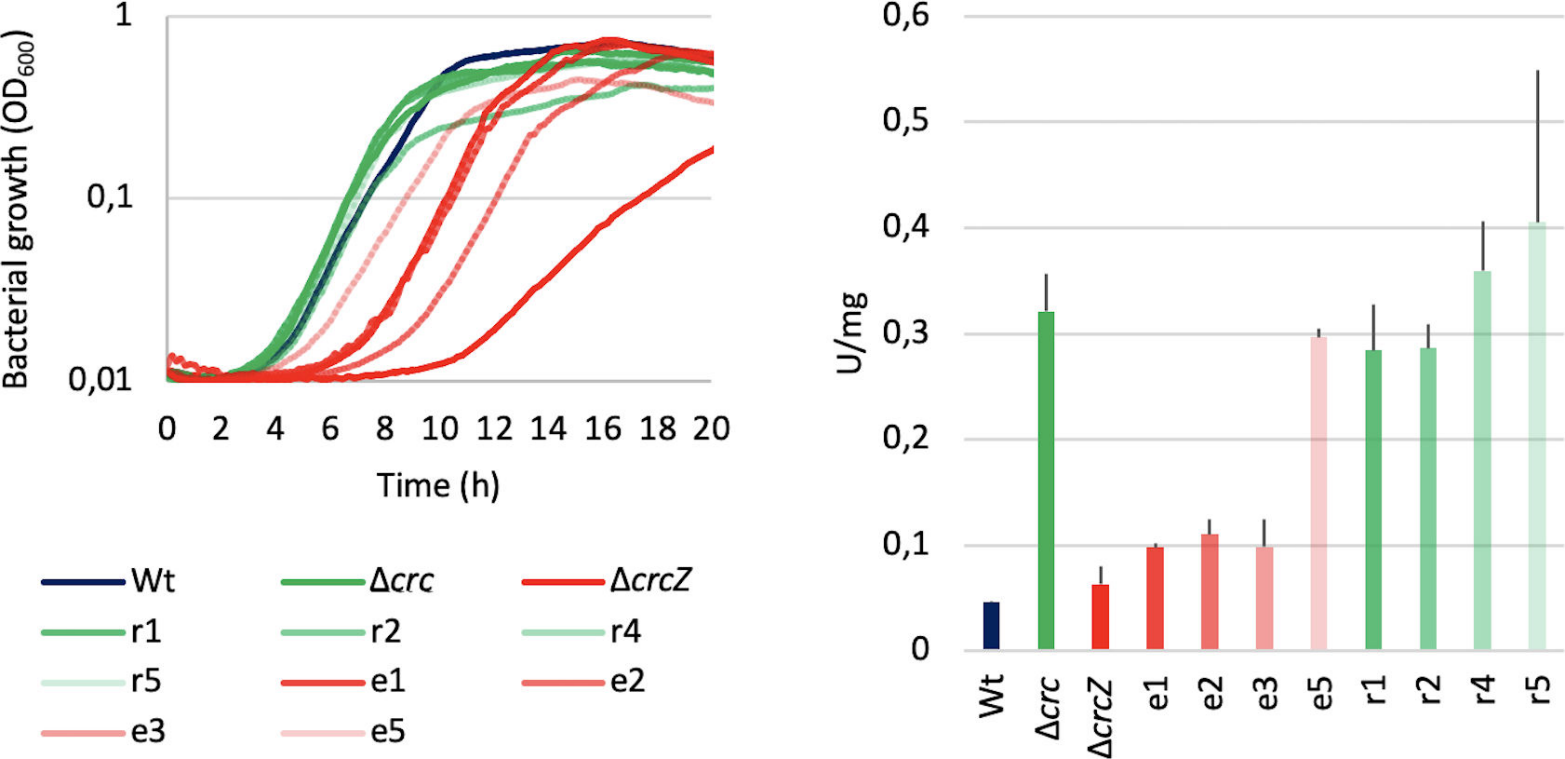
Effect of ∆*crcZ* pseudo-revertants on carbon catabolic repression. (**A**) Growth of the pseudo-revertants in the non-preferred carbon source, citrate. Each curve represents the average of three replicates. (**B**) Glucose-6-phosphate-1-dehydrogenase activity of the pseudo-revertants. Glucose-6-dehydrogenase activity is represented in enzymatic activity units and normalized by the quantity of proteins used in the assay. Experiments were performed in triplicate; error bars represent standard deviation. Asterisk indicates *P* < 0.05 calculated by unpaired two-tail *t*-test. Blue, comparison with the wild-type strain; green, comparison with the ∆*crc* mutant; and red, comparison with the ∆*crcZ* strain.

It is known that Crc regulates *P. aeruginosa* susceptibility to antibiotics and to oxidative stress (17, 18). We thus analyzed the susceptibility to antibiotics and to paraquat of the selected mutants in comparison with the wild-type strain and mutants lacking either *crc* or *crcZ*. As shown, all mutants are more susceptible, although, in different degree, to antibiotics (Table 1) and to paraquat (Fig. 6B) than their parental ∆*crcZ* mutant. In addition, those mutants unable to proficiently grow in FAA using succinate as carbon source, presenting severe defects in catabolite repression control, are even more susceptible than the wild-type strain and resemble the *crc*-defective mutant used as control. In agreement with these data, the mutants presenting strong defects in carbon catabolite repression display a degree of swarming motility similar to that of the *crc* mutant, while the other mutants presented a dendritic pattern and area of swarming more similar to the wild-type strain (Fig. 6A). It is worth mentioning that the pseudo-revertants presenting strong catabolic repression produced high levels of pyocyanin, indicating a concerted action of Crc and CrcZ for this specific phenotype. However, in the case of elastase and pyoverdine, whose expression is impaired in the ∆*crcZ* mutant but not in the ∆*crc* one, both types of pseudo-revertants presented a similar phenotype, restoring the production of these virulence determinants (Fig. 6). Notably, mutants able of growing in FAA partly recovered the virulence of the wild-type *P. aeruginosa* strain, while the mutants presenting strong catabolic repression behave as the *crc*-deficient mutant in a *C. elegans* virulence model (Fig. 6). The main effect of a constitutive catabolite repression phenotype is the incapability of the ∆*crcZ* mutant of growing in non-preferential carbon sources. In agreement with results above discussed, the selected pseudo-revertants presented an improved growth in citrate as compared with their parental ∆*crcZ* mutant, being the effect higher for those mutants able to grow in FAA (Fig. 7A). The glucose-6-phosphate-1-dehydrogenase Zwf is one of the metabolic enzymes under carbon catabolic control in *P. aeruginosa* (9). To further analyze the effect of the observed mutations on the level of carbon catabolite control, we measured the activity of this enzyme in the selected mutants. As shown in Fig. 7B, in the mutants presenting a potential strong defect in catabolite repression, the activity of the enzyme was even higher than that of the wild-type strain, whereas for most of the other mutants, just a small, not statistically significant increase in enzymatic activity was observed. Notably, the only mutant able of growing in FAA and presenting increased levels of Zwf, similar to those of the wild-type strain, was also the only one presenting a mutation in *hfq*.

### Conclusions

The study of spontaneous ∆*crcZ* pseudo-revertant mutants has allowed to make a more precise *in vivo* genetic analysis on the elements that are functionally linked to *P. aeruginosa* carbon catabolite repression. We found that seven out of the eight studied mutants presented mutations located in *crc*, and the last one presented a mutation in *hfq.* Among them, two mutations presumably fully inactivated *crc* (Crc W22stop and Crc G227fs), other two also abolished the phenotype of catabolite repression (Crc 148L and Crc Δ215-234), and the other three mutants presented different degree of catabolite repression, indicating that their mutations impaired Crc activity but do not fully inactivate this repression (Crc A188P, Crc D157N, and Crc T225P). The last strain presented a mutation in *hfq* that rendered, as well, a mild catabolic repression phenotype (Hfq Y55N). The current model of *Pseudomonas* carbon catabolite repression, mainly based on *in vitro* analysis, states that Hfq binds its target mRNAs and Crc stabilizes the ternary complex and increases the affinity of Hfq for this target (23). Our *in vivo* results support this model; when Crc is absent, *P. aeruginosa* does not present catabolite repression, whereas some mutations, likely decreasing Crc of Hfq/mRNA affinity, reduce the constitutive catabolite repression presented by the ∆*crcZ* mutant. These means that, as proposed by *in vitro* studies, Hfq and Crc exert together catabolite repression, and CrcZ is the element that modulates this activity. It is important to notice that a *crc* inactivating mutation is dominant over a *crcZ* inactivating one, even when Hfq is present. This indicates that although it is well established that Hfq is the element able of binding the mRNAs of the genes under carbon catabolite repression, when Crc is absent this repression is not exerted; even when CrcZ is absent and *P. aeruginosa* presents a constitutive hyper-repression phenotype. Hfq is an RNA chaperon ubiquitously distributed among several bacterial species, including those *Escherichia coli* that do not produce Crc (35). In the case of *P. aeruginosa*, it has been recently described that Hfq participates in the regulation of a variety of post-transcriptionally regulated bacterial pathways, besides carbon catabolic repression (20). It can be then expected that Hfq is involved in different regulatory networks and that the specificity of the response comes, at least partly, from its partner, in the case here studied, Crc. Indeed, our results indicate that, although Crc lacks RNA binding activity (11), its inactivation produces similar effects than *hfq* inactivation, at least for the phenotypes analyzed in the present work. Furthermore, this effect is observed in both ∆*crcZ* and wild-type genetic backgrounds, despite that in the ∆*crcZ* mutant, Hfq is not sequestered by CrcZ, and hence, it should be fully active in its capacity for binding its mRNA targets. This indicates that Hfq strictly requires Crc to regulate *P. aeruginosa* catabolic repression. Together with previous publications (12, 17, 18, 36), our results support that Crc has a critical role in *P. aeruginosa* carbon catabolite repression and in linking bacterial metabolism with antibiotic resistance and virulence.

## MATERIALS AND METHODS

### Bacterial strains and growth conditions

Bacterial strains were grown in LB Lennox medium (37) unless otherwise indicated. Ampicillin at 100 µg/mL (for *E. coli*) and carbenicillin at 350 µg/mL (for *P. aeruginosa*) were used when required. Strains and plasmid used in this study are listed in Table 4.

### Construction of a ∆*crcZ* mutant

To construct the ∆*crcZ* mutant, the procedure based on the homologous recombination described by Hoang and coworkers was used (31). Two DNA fragments of 1,000 bp adjacent to the *crcZ* gene were amplified with the primers pairs crcZ_F_HindIII and crcZ_cons_R and crcZ_R_HindIII and crcZ_cons_F. A second PCR with the crcZ_F_HindIII and crcZ_R_HindIII primers was done with the previous PCR products as the template to get a 2,000-bp fragment containing the *crcZ* flaking DNA regions. This amplicon was cloned into the pGEM-T easy vector creating the pGEM-T-Δ*crcZ* plasmid. Then, pGEM-T-Δ*crcZ* was digested with HindIII, and the *crcZ-*flanking DNA fragment introduced into the pEX18Ap plasmid; given rise to the plasmid pEX18Ap-∆*crcZ*. The *E. coli* S-17 conjugative strain was transformed with the resultant plasmid, which was introduced into *P. aeruginosa* PAO1 by conjugation. Briefly, 100 µL of an overnight culture of the conjugative strain *E. coli* S-17 and 100 µL of a 0.6 OD culture of *P. aeruginosa* incubated 2 h at 42°C were mixed and washed twice with LB and then seeded onto a LB plate. After 20 h of incubation, *P. aeruginosa* mutants with the pEX18Ap- ∆*crcZ* plasmid were selected in LB with 350 µg/mL carbenicillin and 5% (wt/vol) sucrose. Clones presenting Cb^R^ and Sac^S^ phenotypes were streaked in LB with 5% (wt/vol) sucrose and grown during 48 h at 30°C. Then the Cb^S^ and Sac^R^ were checked again, and the deletion of the *crcZ* gene was confirmed by PCR using the crcZ_F_HindIII and crcZ_R_HindIII and the crcZ_ver_F and crcZ_ver_R primers. Primers used in this work are shown it Table 5.

### Experimental evolution and isolation of *crcZ* pseudorevertants

Five 100-mL flasks, each one containing 20 mL of LB, were seeded with the ∆*crcZ* mutant strain from the −80°C stored stock and were incubated at 37°C with shaking at 250 rpm. Each day, 24 h since the initial inoculum, 20 µL of each culture was transferred to a new flask (dilution 1:1,000) during 10 d.

Appropriate dilutions (10^−7^, 10^−8^) of the 24-h grown cultures were seeded on LB plates to isolate colonies of each culture. Twenty colonies of each day were checked to evaluate their phenotype concerning catabolite repression by using the method described by Collier and coworkers (34). Succinate exerts catabolite repression on the AmiE translation. AmiE transforms enzymatically the non-toxic compound FAA into fluoroacetate, which is toxic when it is transformed into fluorocitrate (34). Then, strains of ∆*crcZ* that lack catabolite repression do not growth in M63 succinate in presence of FAA 5 mg/mL. Twelve strains of the sixth subculture day, six with and six without catabolite repression, were picked and stored for subsequent analysis.

### Genomic DNA purification and sequencing

Genomic DNA purification and quality analysis were performed by the Translational Genomics Unit (Instituto Ramón y Cajal de Investigación Sanitaria-Hospital Ramón y Cajal from Madrid). DNA was extracted using the Chemagic DNA Bacterial Kit H96 (CMG-799 Chemagic) and the equipment Chemagic 360/MSMI (PerkinElmer). DNA quality was determined using an Agilent 2200 TapeStation System. The construction of pair-end libraries (2 × 150) and whole-genome sequencing were performed by the Oxford Genomics Centre using an Illumina NovaSeq6000 system. Coverage was higher than 150× for all samples.

Obtained paired-end reads were mapped to the *P. aeruginosa* PAO1 reference genome (GenBank accession: NC_002516.2) with Bactmap v1.0 to create the variant calling files (38). BCFtools were used for filtering and binding individual variant calling files (39).

### Phenotypic characterization of the selected clones

For growth in different carbon sources, overnight cultures in LB of the bacterial cells were washed twice with minimal M63 medium without any carbon source. Ten microliters of cells at the appropriate OD_600_ were used to inoculate microtiter plates with 140 µL of each of the media used in the analysis, namely, 40 mM succinate, 40 mM gluconate, 40 mM citrate, or 40 mM mannitol, in each well at OD_600_ 0.01: LB-Lennox (Pronadisa), Mueller-Hinton (MH) (Pronadisa), and M63 (USBiochemical). Bacteria were grown at 37°C with intermittent shaking, and growth was recorded by measuring the OD_600_ every 10 min in a Tecan Infinite M200 plate reader.

#### Susceptibility to antibiotics

The minimal inhibitory concentrations (MICs) of colistin, fosfomycin, ceftazidime, meropenem, aztreonam, ofloxacin, chloramphenicol, polymyxin B, imipenem, tetracycline, tobramycin, streptomycin, amikacin, kanamycin, and gentamycin for the selected strains were determined using Epsilon-test (E-test) strips (Biomerieux). One hundred microliters of a 0.85% NaCl bacterial suspension with OD_600_ 0.005 were seeded onto MH plates. MIC was recorded after 20 h of growth at 37°C.

#### Susceptibility to oxidative stress

Bacterial cells were grown in the same way as aforementioned. Discs containing 1 µmol of paraquat were used instead E-test strips. The inhibition halo of growth was measured.

#### Virulence

Virulence testing of the *P. aeruginosa* strain was done with *Caenorhabditis elegans* Bristol N2 by the slow killing procedure described in the references (40, 41) with some modifications. *C. elegans* were grown for their maintenance on potato dextrose agar (PDA) plates (Oxoid) seeded with *E. coli* OP50 at 20°C. The strains to be analyzed were grown during 20 h at 37°C in 6-cm PDA plates. Five females of *C. elegans*, obtained from the *E. coli* seeded plates, were dropped in each plate, and the number of worms was recorded each day along 5 d.

#### Motility

Swarming motility assays of *P. aeruginosa* were done using LB plates containing agar at 0.5% . Five microliters of an overnight culture of each of the analyzed strains were used for the inoculation, and the plates were grown at 30°C during 20 h in a sealed box to preserve the humidity.

#### Pyocyanin

For analyzing pyocyanin production, 1 mL of bacterial culture was centrifuged (7,000 rpm, 3 min), and the supernatant was filtered through a 200-nm pore filter (Whatman). The amount of pyocyanin was estimated by measuring the absorbance of the obtained supernatants at 690 nm in microtiter plate reader (Tecan Infinite 200 m). Triplicate cultures were used in all cases.

#### Elastase

*Pseudomonas aeruginosa* cells were cultured in 20 mL of LB at 37°C for 24 h. At the late stationary phase, 2-mL samples were collected from each culture and centrifuged for 10 min at 7,000 rpm, and the supernatants were filtered using 0.2-µm filters (Whatman). The elastase assay was adapted from Kessler and Safrin (42); 1 mL of Congo Red elastin (Sigma-Aldrich) was added to 100 µL of each sample, and the mixture was incubated at 37°C and 250 rpm for 2 h. Subsequently, samples were centrifuged (10 min, 7,000 rpm), and the OD 495 nm of the filtered supernatants was determined. Three replicates of each condition were included in the analyses.

#### Pyoverdine

*Pseudomonas aeruginosa* cells were cultured in 20 mL of LB at 37°C for 24 h. Pyoverdine production was measured using the method described by Hoegy et al. (43). Overnight cultures were diluted in a 1:10 ratio with 50 mM Tris–HCl at pH 8. The samples were placed in a 96-well plate, and the fluorescence emitted at 447 nm was measured after exciting the sample to a 400-nm wavelength using a TECAN Spark multiplate reader.

### Glucose-6-phosphate dehydrogenase activity

Bacteria were grown in LB until exponential growth phase (OD_600_ ≈0.6) was reached, and 10 mL of each of the cultures was centrifuged (7,000 rpm; 3 min; 4°C). The pellets were suspended in phosphate buffer (50 mM; pH 7.5) and sonicated as above described. Glucose-6-phosphate dehydrogenase activity was measured as described (18). Ten microliters of the cellular extract were used in a final volume of 100 µL. The reaction mixture contained 50 mM phosphate buffer pH 7.5; 10 mM MgSO4, 0.75 mM NADP+; and 2 mM glucose-6-phosphate. NADPH production was measured by spectrophotometry at 340 nm. Extinction coefficient of NADPH (6.22 mM^−1^ min^−1^) was used to calculate enzymatic activity, and the results were normalized in function of the protein quantity of the extract.

### Molecular modelling

PDB entry 6o1k was used as the basis to predict and analyze mutant structures of the 2:2:2 complex of Hfq, amiE, and Crc (composed of two Hfq hexamers, two *amiE* molecules, and two Crc molecules) (22). Complete structures of the mutants were predicted using Modeller (44) through the UCSF Chimera (45) user interface, except for the Crc mutant Δ*215-234* which was generated using I-TASSER (46). The mutant proteins were used to substitute their counterparts in the reference complex to obtain initial 2:2:2 mutant complexes. CHARMM-GUI (47) was used to generate the systems needed for simulation: the wild-type and mutant complexes were solvated in 0.15 m KCl using a box with a margin of 10 Å to either side of the complex placing ions using a Monte-Carlo method.

All complexes were minimized and subsequently equilibrated in the NVT ensemble at 303.15°K prior to a production MD simulation in the NPT ensemble at 303.15 °K and 1 atm employing throughout the CHARMM36m force field and periodic boundary conditions. The production run was initially calculated for 10 ns and then continued for an additional 100ns using GROMACS (48).

Complex structures before and after the simulation were compared using UCSF Chimera to calculate the number of H-bonds and atomic contacts between the Hfq hexamers and the *amiE* RNA, the Hfq hexamers and the Crc dimer, and both molecules of the Crc dimer and the Crc dimer and the *amiE* RNA.

## Data Availability

The sequences analyzed in this work can be found at NCBI BioProject ID (PRJNA934266). All other data are included in the paper.
